# Phase-amplitude coupling-based adaptive filters for neural signal decoding

**DOI:** 10.3389/fnins.2023.1153568

**Published:** 2023-05-02

**Authors:** Jiajun Li, Yu Qi, Gang Pan

**Affiliations:** ^1^State Key Lab of Brain-Machine Intelligence, Zhejiang University, Hangzhou, China; ^2^College of Computer Science and Technology, Zhejiang University, Hangzhou, China; ^3^Affiliated Mental Health Center and Hangzhou Seventh Peoples Hospital, MOE Frontier Science Center for Brain Science and Brain-Machine Integration, Zhejiang University School of Medicine, Hangzhou, China

**Keywords:** electrocorticography (ECoG), phase-amplitude coupling, deep learning, band-pass filter, brain-computer interface

## Abstract

Bandpass filters play a core role in ECoG signal processing. Commonly used frequency bands such as alpha, beta, and gamma bands can reflect the normal rhythm of the brain. However, the universally predefined bands might not be optimal for a specific task. Especially the gamma band usually covers a wide frequency span (i.e., 30–200 Hz) which can be too coarse to capture features that appear in narrow bands. An ideal option is to find the optimal frequency bands for specific tasks in real-time and dynamically. To tackle this problem, we propose an adaptive band filter that selects the useful frequency band in a data-driven way. Specifically, we leverage the phase-amplitude coupling (PAC) of the coupled working mechanism of synchronizing neuron and pyramidal neurons in neuronal oscillations, in which the phase of slower oscillations modulates the amplitude of faster ones, to help locate the fine frequency bands from the gamma range, in a task-specific and individual-specific way. Thus, the information can be more precisely extracted from ECoG signals to improve neural decoding performance. Based on this, an end-to-end decoder (PACNet) is proposed to construct a neural decoding application with adaptive filter banks in a uniform framework. Experiments show that PACNet can improve neural decoding performance universally with different tasks.

## 1. Introduction

Electrocorticography (ECoG) has been widely used for clinical diagnoses and brain-computer interfaces (BCIs) (Wolpaw et al., 2002; Miller et al., 2010a). Compared with electroencephalography (EEG), which mostly reflects spectral activities below 50 Hz, ECoG contains high-frequency activities (up to 200 Hz), which encodes rich information for motor and cognitive behaviors. The rich information in the ECoG signals has enabled various applications, such as the decoding of motor behaviors (Pan et al., 2018; Wang et al., 2018; Xie et al., 2018; Qi et al., 2022) and synthesis of spoken words or sentences (Chakrabarti et al., 2015), demonstrating great potential in rehabilitation and neuroprosthesis (Miller et al., 2010b).

To extract frequency domain features in ECoG signals, bandpass filters are widely used to focus on the neural activity in a certain frequency band. Commonly used frequency bands such as delta (0.1–4 Hz), theta (4–8 Hz), alpha (8–12 Hz), beta (12–30 Hz), and gamma (above 30 Hz) bands can reflect the normal rhythm of the brain. However, the universally predefined bands might not be optimal for a specific task. Especially the gamma band usually covers a wide frequency span (i.e., 30–200 Hz). Existing studies further divide the gamma band into low-gamma (30–70 Hz) and high-gamma (70–200 Hz), however, it is also too coarse to capture features that appear in narrow bands. Studies have shown that the sub-frequency bands in the gamma band contain useful information (Miller et al., 2009). Therefore, how to make better use of the abundant high-frequency components in ECoG signals is a very important but thorny problem. To select informative frequency bands from the high-gamma band, efforts have been made in previous studies. One widely used way to locate useful frequency bands in ECoG signals is brute force search. These decoders usually use Fourier transform or wavelet transforms to extract the power of certain frequency bands as the features and evaluate the features in a task-driven manner to select the effective narrow bands (Yanagisawa et al., 2011; Chestek et al., 2013; Bleichner et al., 2016; Branco et al., 2017; Li et al., 2017; Zhu et al., 2021; Qi et al., 2019). The other widely used way is using end-to-end decoders via deep learning technology. Deep learning decoders have recently gained prevalence in ECoG signal studies due to their capacity to directly learn with raw data. These decoders, such as CNNs and RNNs have a strong learning ability to automatically locate the effective frequency domains (Pan et al., 2018; Xie et al., 2018). The last few years have seen the emergence of some very efficient deep learning models for EEG signals, such as EEGNet (Lawhern et al., 2018), DeepConvNet, and ShallowConvNet (Schirrmeister et al., 2017). Although deep learning models have made progress in designing end-to-end brain signal decoders, these methods still have some limitations. On the one hand, the training of deep models requires a large amount of data which is usually impractical for brain signals; on the other hand, frequency band selection using these models mostly lacks explainability.

From the perspective of neuroscience, studies have shown the coupled working mechanism of synchronizing neurons and pyramidal neurons in neuronal oscillations (Bragin et al., 1995; Canolty et al., 2006; Canolty and Knight, 2010), which can provide guidance for effective frequency band selection. Miller et al. (2010a) proposed that rhythms may also play a role in suppressing local cortical computation, with the cortically suppressed (disengaged) state one in which widespread populations of cortical neurons are phase-coupled to the rhythm. These neuronal mechanisms, collectively known as Cross-frequency coupling (CFC) is widely used to coordinate neural dynamics across spatial and temporal scales (Bleichner et al., 2016), but both types of decoders ignored this mechanism. There are three forms of coupling neural dynamics: phase-amplitude coupling (PAC; Canolty et al., 2006), phase-phase coupling (PPC; Canolty et al., 2007), and amplitude-amplitude coupling (AAC; Bruns and Eckhorn, 2004; Voytek et al., 2010). Especially PAC, where the phase of the low-frequency component modulates the amplitude of the high-frequency activity, plays an important role in neural information processing and cognition, for example, in learning and memory.

These findings inspire us to ask whether PAC can help find influential frequency bands adaptively in a task-specific way? In this paper, we propose a PAC-based adaptive band filter for neural signals. Leveraging the PAC mechanisms of synchronizing oscillations, in which the phase of slower oscillations modulates the amplitude of faster ones, we can use low-frequency bands to locate the sub-frequency bands from the high-gamma range in a task-specific and individual-specific way. Thus, the information can be more precisely extracted from ECoG signals to improve neural decoding performance. Based on this, an end-to-end decoder (PACNet) is proposed to construct a neural decoding application with adaptive filter banks in a uniform framework. The proposed approach achieves higher decoding performance improvement over previous decoders.

## 2. Materials and methods

**Figure 1:**
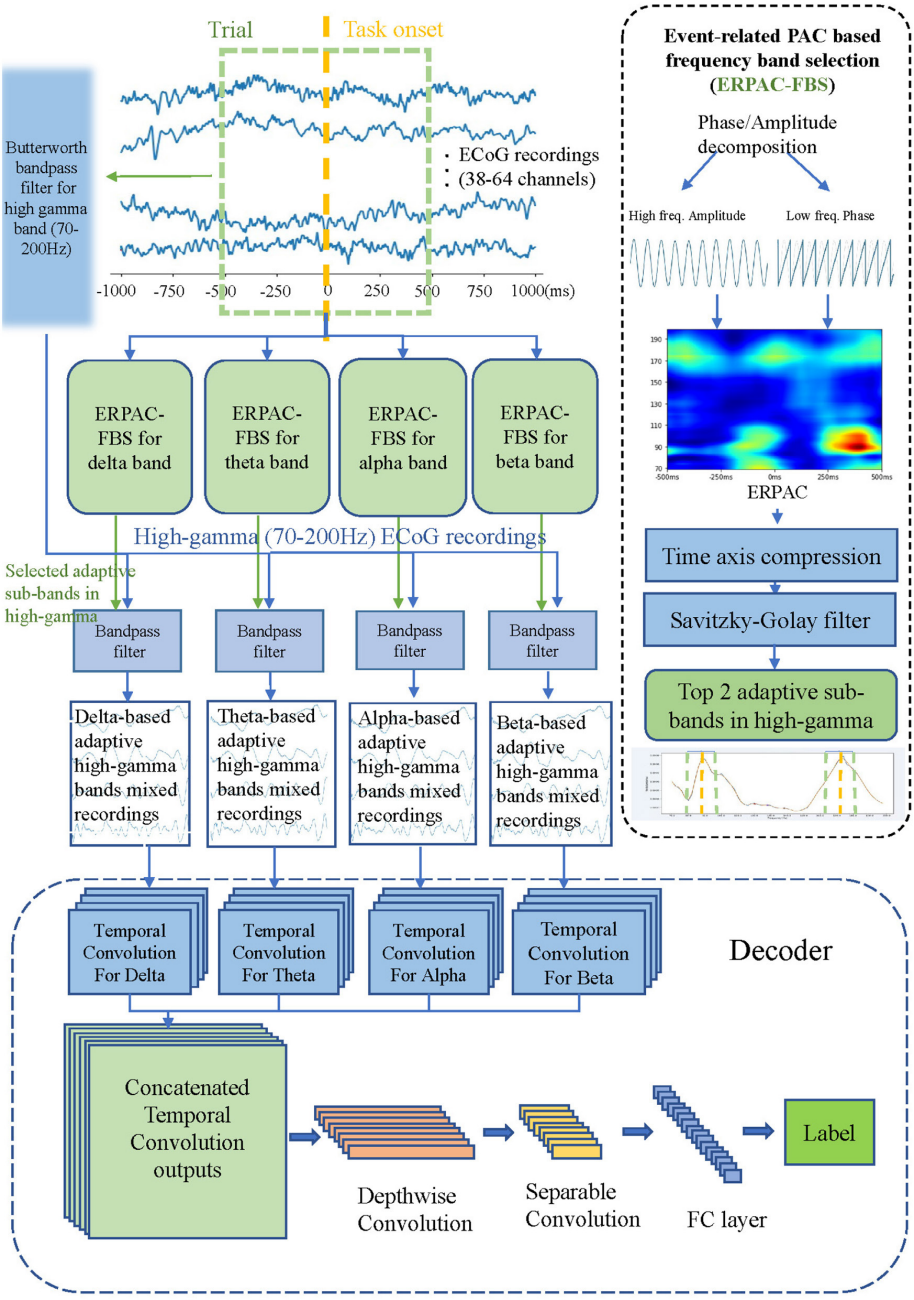
Framework of the PACNet, including the flow chart of our kernel module: Event-related PAC based frequency band selection algorithm (ERPAC-FBS) and the proposed deep network structure. First, we use ERPAC-FBS to get two best sub-bands in the high-gamma range based on each low-frequency band, and then these two sub-bands are used to carry out band-pass filtering on the high-gamma signal, and the corresponding target signal is obtained. Finally, we put these mixed corresponding target signals into the proposed decoder.

Here we will present the proposed method in detail. Firstly, we will introduce the dataset we used and the preprocessing method. Then we will present our data-driven band filter in detail. Finally, we propose the PACNet that constructs a neural decoding application with PAC-based filter banks in a uniform framework. Figure 1 shows framework of the proposed method, including the flow chart of our kernel module: Event-related PAC based frequency band selection algorithm which is represented in Section 2.2 and PACNet in Section 2.3. We will open our source core code of Event-related PAC based frequency band selection algorithm and PACNet on GitHub, our code can be found at: https://github.com/PuddingZJU/PACNet.

### 2.1. Electrocorticography dataset

Two publicly available ECoG datasets are adopted in our study, including a motor task and a visual task. These public data can be found at Miller (2019).

#### 2.1.1. Motor dataset

We used KJM's FingerFlex dataset (Miller et al., 2012), which is based on finger movement. During the finger movement task, subjects were cued with a word displayed on a bedside monitor indicating which finger to move during 2 s movement trials. The subject performed self-paced movements in response to each of these cues, and they typically moved each finger 2–5 times during each trial, but some trials included many more movements. A 2 s rest trial (blank screen) followed each movement trial. There were 30 movement cues for each finger, and trials were interleaved randomly. Finger positions were recorded using a 5-degree-of-freedom dataglove sensor (5 dt, Irvine, CA). Since the original data is a study on the fitting of finger bending trajectory, we here transform it into a classification problem of finger motion through the maximum threshold of bending, that is, which finger moves in this movement. This is a five-classification problem, so we need the decoding performance of multi-classification in the test period. Through the pre-processing of the data, we finally found that there were nine subjects in the data set, and the total number of effective trials reached 7,150, among which 406 were the subjects with the least trial and 1,682 were the subjects with the most.

#### 2.1.2. Visual dataset

Face-House discrimination task Subjects performed a basic face and house stimulus discrimination task (Miller et al., 2016). They were presented with grayscale pictures of faces and houses (luminance- and contrast-matched) that were displayed in random order for 400 ms each, with 400 ms blank screen inter-stimulus interval (ISI) between the pictures. The 10 cm-wide pictures were displayed at 1 m from the patients while they were seated at the bedside. There were three experimental runs with each patient, with 50 house pictures and 50 face pictures in each run (for a total of 300 stimuli). In order to maintain fixation on the stimuli, patients were asked to verbally report a simple target (an upsidedown house), which appeared once during each run (1/100 stimuli). There were a few errors in reporting the upside-down target house in any run (~2–3 across all 21 experimental runs).

### 2.2. PAC-based adaptive band filter design

#### 2.2.1. Event-related PAC based frequency band selection

Here we divide the ECoG signal into two components: low-frequency narrowband signal and high-frequency broadband signal. Based on previous researches (Miller et al., 2010a), low-frequency narrowband signals contain five bands: δ(1–3 Hz), θ (4–8 Hz), α (8–12 Hz), β (12–20 Hz) and “*Canonical γ*” (30–50 Hz). While the high-frequency broadband frequency band (above 50 Hz) has not been finely divided. We propose a Phase-amplitude coupling (PAC)-based method to infer the relevant high-frequency band from the known low-frequency band for adaptive filtering of ECoG signal, and combine some frequency bands with similar carriers (low-frequency bands) to synthesize a new composite frequency band signal, to act as the band filter.

##### 2.2.1.1. Computing of instantaneous phases and amplitudes

The first step is to extract instantaneous phases from low-frequency bands and amplitudes from high-frequency signals. Specifically, we use the Hilbert transform and a bandpass Butterworth filter to separate the original signal low-frequency phase signal and high-frequency amplitude signal from the original data. First we put *x*_*raw*_[*t*] into bandpass Butterworth filters, to generate low-frequency time series *x*_δ_[*t*], *x*_θ_[*t*], *x*_α_[*t*], *x*_β_[*t*] and high frequency time series *x*_γ_[*t*]. Then the Hilbert transform (Pilcher and Rusyniak, 1993)


(1) $$y[t]=H(x[t])=\frac{1}{\pi }\int_{\infty }^{-\infty }\frac{x[\tau ]}{t-\tau }$$

is applied to each filtered time series to convert the cosine wave into a sine wave which delays the original signal by $\frac{\pi }{2}$ at each frequency band. After that, we can compute the instantaneous phase by the original and transformed signal. Finally, we can apply the transform on two paired low-high frequency time series to make them become analytic signals, for example, we compute alpha band and high-gamma band signals as


(2) $$\begin{array}{l} z_{\alpha }\left[t\right]=x_{\alpha }\left[t\right]+iy_{\alpha }\left[t\right]=a_{\alpha }\left[t\right]e^{i\phi_{\alpha }\left[t\right]} \end{array}$$

(3) $$\begin{array}{l} z_{\gamma }\left[t\right]=x_{\gamma }\left[t\right]+iy_{\gamma }\left[t\right]=a_{\gamma }\left[t\right]e^{i\phi_{\gamma }\left[t\right]} \end{array}$$

where ϕ_α_[*t*] and ϕ_γ_[*t*] are the instantaneous phases, and *a*_α_[*t*] and *a*_γ_[*t*] are the instantaneous amplitudes of the *X*_α_[*t*] and *X*_γ_[*t*] time series. These instantaneous phases and amplitudes are used to compute phase-amplitude coupling.

##### 2.2.1.2. Computing of event-related PAC

Most techniques for calculating PAC provide a numerical index that represents an average value across an arbitrarily long time period. But our datasets are usually event-based and need to respond to mutation events. Here we use event-related phase/amplitude coupling (ERPAC) (Voytek et al., 2013) designed to capture the temporal evolution of task-related changes in PAC across events or between distant brain regions that is applicable to human or animal electromagnetic recording. The ERPAC is based on a circular-linear correlation (Zar, 1999) which evaluates the Pearson correlation, across trials, of the amplitude α_*t*_ and with the sine and cosine of the phase ϕ_*t*_. We denote by *c*(*x, y*) the Pearson correlation between two variables *x* and *y*, *r*_*sx*_ = *c*[*sin*(ϕ_*t*_), α_*t*_], *r*_*cx*_ = *c*[*cos*(ϕ_*t*_), α_*t*_], and *r*_*sc*_ = *c*[*sin*(ϕ_*t*_), *cos*(ϕ_*t*_)] hence, the circular-linear correlation *p*_*cl*_ is defined as


(4) $$\begin{array}{l} p_{cl}=\sqrt{\frac{r_{sx}^{2}+r_{cx}^{2}-2r_{sx}r_{cx}r_{sc}}{1-r_{sc}^{2}}} \end{array}$$

Thus, we can obtain PAC significance on the time scale. We used the implementation of ERPAC in tensorpac (Combrisson et al., 2020). After that, we get a matrix representing the PAC intensity in the temporal dimension, in order to select the effective frequency band, we use the maximum PAC intensity of each frequency in the temporal dimension to turn the matrix into a one-dimensional vector. Then we use the Savitzky-Golay filter to compute the local maximum value as the effective frequency, We sorted the intensity values of these frequency points, taking the frequency with high intensity as the center point of the target frequency band first, and finally taking these center points plus width *BW* as the effective frequency band, the value of *BW* we used is 10.

The event-related phase-amplitude coupling-based frequency band selector is presented in Algorithm 1. According to the previous work (Aru et al., 2015), if Δ*f*_2_ <2*f*_1_, it will cause false negative results. We drop the “*Canonical γ*,” and just use four low-frequency bands to calculate the results. After obtaining the optimal high-frequency band corresponding to each low-frequency band through screening, we first apply band-pass filtering to the original signal using each sub-high-frequency band and then superimpose them together to obtain the most relevant mixed high-frequency band signal in this low-frequency band. However, there is a question of whether the original low-frequency signal should be added to the newly obtained mixed-frequency signal. At present, we have not found a fixed answer, so we conducted experiments in both cases to verify the role of low-frequency signals in the decoding process.

**Algorithm 1:** Event-related phase-amplitude coupling based frequency band selection.

**Input:** Input single channel raw signal vector *x* containing *N* trials, the shape of *x* is (*N, samplerate*×*time*).
**Output:** A list of mixed adaptive sub-bands in high-gamma band named as *Band*_*result*
1: Step 0: Initialization
2: Initialize *Erpac*_*results* as an empty list
3: Initialize *Band*_*results* as an empty list
4: Step 1: Calculate Event-related phase-amplitude coupling of each low-frequency band (*LB*) phase with high-gamma band amplitude
5: for each *LB* in [*delta, theta, alpha, beta*] **do**
6: *PAC*_*Weight*← EventRelatedPac(*x*, phase_band = *LB*, amplitude_band = high-gamma )
7: Put *PAC*_*Weight* into *Erpac*_*results* for temporary storage
-*Step*2:*Frequencybandselection*
8:9: each *E* in Erpac_result
10: *E* is two-dimensional matrix (*time*×*frequency*), compress the time axis using the mean, got a vector on frequency axis *f*
11: use Savitzky-Golay filter to calculate local maximum value of *f* named as *f*_*peaks*
12: sort *f*_*peaks* by its *f* value, the order in which the larger the value is, the higher the priority is, finally chose top 2 peaks.
13: for each *p* in *f*_*peaks* **do**
14: *best*_*band*← [*p*-10,*p*+10]
15: Put *best*_*band* into *Band*_*results* for storage
-
16:17: return Band_results

### 2.3. PACNet: a deep neural network architecture with the PAC-based band filter

PACNet is an example of integrating this filter with existing deep-learning decoders. Here, the basic deep learning model we choose is EEGNet, a trendy model. Its advantages are compact design, few parameters, and less demand for computing resources. On the other hand, EEGNet can process Raw data without manually extracting features, such as power spectrum in the frequency domain, which reduces information loss. At the same time, it can better reflect the impact of input data noise on the deep learning network decoder, indicating the role of the filter we designed (Lawhern et al., 2018). For PACNet, As mentioned above, the input data became four signals of different frequency bands after filtering, which were independent of each other. Therefore, we divided the first sequential winding layer into four layers and carried out the separate one-dimensional winding operation on the four signals to extract independent features to prevent mutual interference between different frequency bands. We have represented that PACNet is an end-to-end decoder proposed to construct a neural decoding application with adaptive filter banks in a uniform framework. So PACNet can have more than one version introduced in this paper. It can quickly build another PACNet based on other decoders with an independent temporal module and split them into four parts for four signals of different frequency bands after filtering, and we think this is a uniform framework. The PACNet is developed using Python 3.9 and Tensorflow 2.5, and runs on a computer with NVIDIA GeForce RTX 3090 graphics cards. We will release PACNet based on other models in the future on our GitHub repository.

## 3. Results

### 3.1. Neural decoding performance and comparison

In this section, we will introduce the process and results of our decoding performance test on filter and PACNet in detail. First, we detail the existing algorithms and test methods we compare. After that, we present and analyze the results.

#### 3.1.1. Approaches in comparison

To reveal the performance of adaptive band filters and PACNet, we used 100 times repeated k-fold cross-validations and paired *t*-test for each competitor on the ECoG dataset to test a decoder that has been popular recently. Because our filter is based on the filtering of raw data, the decoder that can best reflect its effectiveness is the one that can directly process raw data, so we choose the currently commonly used deep learn-based decoder for a comparison test, and the decoders for comparison including three representative neural network-based models of EEGNet (Lawhern et al., 2018), DeepConvNet, and ShallowConvNet (Schirrmeister et al., 2017). The common feature of these three decoders is that they do not need to extract features in advance and can directly accept raw data input for feature extraction and classification of signals.

We tested the approaches with two data sets, one from the motor task and the other from the visual task. We all use all data sets with the subject of the test method. In each type of data on the classification test, the test accuracy calculation method adopted 10-fold cross-validation 100 times. Then we used paired *t*-test for each competitor to evaluate the significance of the results, and our only hypothesis is that our model has better decoding performance than other models. In 10-fold cross-validation, the data according to the label equal proportion randomly into 10 portions, take turns to take nine serving for training and validation steps, 1 for testing steps, turns 10 times. The final accuracy value is the average result of the 10 training tests 100 times.

#### 3.1.2. Performance and analysis

The overall results are shown in Table 1, and the results of each subject are shown in Tables 2, 3. Table 2 represents the results of the accuracy in fingerflex dataset. Compared with the EEGNet, our method is improved by about 6.2%. Compared with the DeepConvNet, the accuracy of our method is improved by about 9.4%. Compared with the ShallowConvNet, The accuracy of our method is improved by about 11.4%. In paired *t*-test, the overall p-value is smaller than 0.05, so the improvement of our method compared to other methods is significant.

**Table 1:** Overall decoding performance on fingerflex dataset and facehouse dataset.

	**Fingerflex dataset**		**Facehouse dataset**	
**Methods**	**Accuracy**	***P*** **-value**	**Accuracy**	***P*** **-value**
Ours	0.6258 ± 0.1502	–	0.9781 ± 0.0184	–
EEGNet	0.5678 ± 0.1402	0.0170^**^	0.9390 ± 0.0338	0.0104^**^
DeepConvNet	0.5315 ± 0.1612	0.0004^***^	0.9476 ± 0.0301	0.0243^**^
ShallowConvNet	0.5118 ± 0.1699	0.0008^***^	0.9507 ± 0.0455	0.0750^*^

^*^P < 0.05, ^**^0.01 <*P* < 0.05, ^***^P < 0.01.

**Table 2:** Decoding performance on fingerflex dataset of each subject, the best results in each domain are bolded.

**Subject ID**	**Ours**	**EEGNet**	**DeepConvNet**	**ShallowConvNet**
1	**0.5391** **±** **0.0204**	0.5184 ± 0.0135	0.4813 ± 0.0157	0.4463 ± 0.0140
2	**0.4772** **±** **0.0052**	0.4592 ± 0.0061	0.3800 ± 0.0065	0.3653 ± 0.0063
3	0.7716 ± 0.0093	0.7782 ± 0.0060	0.7709 ± 0.0041	**0.7796** **±** **0.0078**
4	**0.5657** **±** **0.0110**	0.4992 ± 0.0107	0.4356 ± 0.0081	0.3654 ± 0.0081
5	**0.4605** **±** **0.0046**	0.4537 ± 0.0061	0.3896 ± 0.0063	0.3900 ± 0.0057
6	**0.7581** **±** **0.0154**	0.6135 ± 0.0261	0.6175 ± 0.0240	0.5218 ± 0.0122
7	**0.8633** **±** **0.0080**	0.6834 ± 0.0113	0.7131 ± 0.0124	0.7490 ± 0.0374
8	**0.4870** **±** **0.0161**	0.3930 ± 0.0095	0.3334 ± 0.0105	0.3467 ± 0.0101
9	0.7098 ± 0.0163	**0.7120** **±** **0.0121**	0.6617 ± 0.0158	0.6425 ± 0.0091
Average	**0.6258** **±** **0.0118**	0.5678 ± 0.0113	0.5315 ± 0.0115	0.5118 ± 0.0123

**Table 3:** Decoding performance on facehouse dataset of each subject, the best results in each domain are bolded.

**Subject ID**	**Ours**	**EEGNet**	**DeepConvNet**	**ShallowConvNet**
1	**0.9820** **±** **0.0014**	0.9180 ± 0.0054	0.9373 ± 0.0016	0.9245 ± 0.0029
2	**0.9915** **±** **0.0020**	0.8927 ± 0.0053	0.9175 ± 0.0050	0.8787 ± 0.0052
3	**0.9725** **±** **0.0015**	0.9372 ± 0.0048	0.9195 ± 0.0008	0.9703 ± 0.0017
4	0.9640 ± 0.0011	0.9573 ± 0.0047	**0.9693** **±** **0.0033**	0.9677 ± 0.0025
5	0.9618 ± 0.0057	0.9550 ± 0.0055	0.9710 ± 0.0035	**0.9732** **±** **0.0025**
6	**0.9867** **±** **0.0027**	0.9662 ± 0.0026	0.9803 ± 0.0031	0.9712 ± 0.0013
7	**0.9885** **±** **0.0016**	0.9467 ± 0.0054	0.9388 ± 0.0028	0.9700 ± 0.0011
Average	**0.9781** **±** **0.0023**	0.9390 ± 0.0048	0.9477 ± 0.0029	0.9508 ± 0.0024

We also conducted experiments with the facehouse dataset, the results are in Table 3, compared with EEGNet, DeepConvNet, and ShallowConvNet, the performance improvement is about 3.9, 3.1, and 2.8%, respectively. In paired *t*-test, the results show the improvement of our method compared to other methods is significant.

#### 3.1.3. Ablation experiments on fingerflex dataset

In order to verify the influence of the PAC filter on the results, we designed an ablation experiment using 2 PACNets with consistent parameters for training and testing, one of which removed the adaptive filter, which means it only had high-gamma (70–200 Hz) band, to test its influence on the network, we used 10-fold cross-validation for each subject of the fingerflex dataset with more than 7,000 trials in total. The final test results are shown in Table 4. Our method has about 2% higher than the control group in the total average value. In the test of a single subject, the vast majority of the test results are higher accuracy using our data-driven filter. So our adaptive filter is adequate. In paired *t*-test, the *p*-value is 0.0068, which shows that the improvement of the PAC filter compared to the non-PAC filter is significant.

**Table 4:** Ablation experiment on fingerflex dataset.

**Subject ID**	**With PAC**	**Without PAC**
1	0.6009 ± 0.0566	0.5885 ± 0.0850
2	0.5005 ± 0.0357	0.4824 ± 0.0301
3	0.7918 ± 0.0482	0.7763 ± 0.0325
4	0.6251 ± 0.0362	0.6219 ± 0.0486
5	0.4672 ± 0.0373	0.4696 ± 0.0206
6	0.8073 ± 0.0933	0.7642 ± 0.0528
7	0.8867 ± 0.0363	0.8771 ± 0.0289
8	0.5413 ± 0.0625	0.5115 ± 0.0716
9	0.7432 ± 0.0439	0.7387 ± 0.0361
Average	0.6627 ± 0.0500	0.6478 ± 0.0451

### 3.2. Analysis of the PAC-based frequency band selection

**Figure 2:**
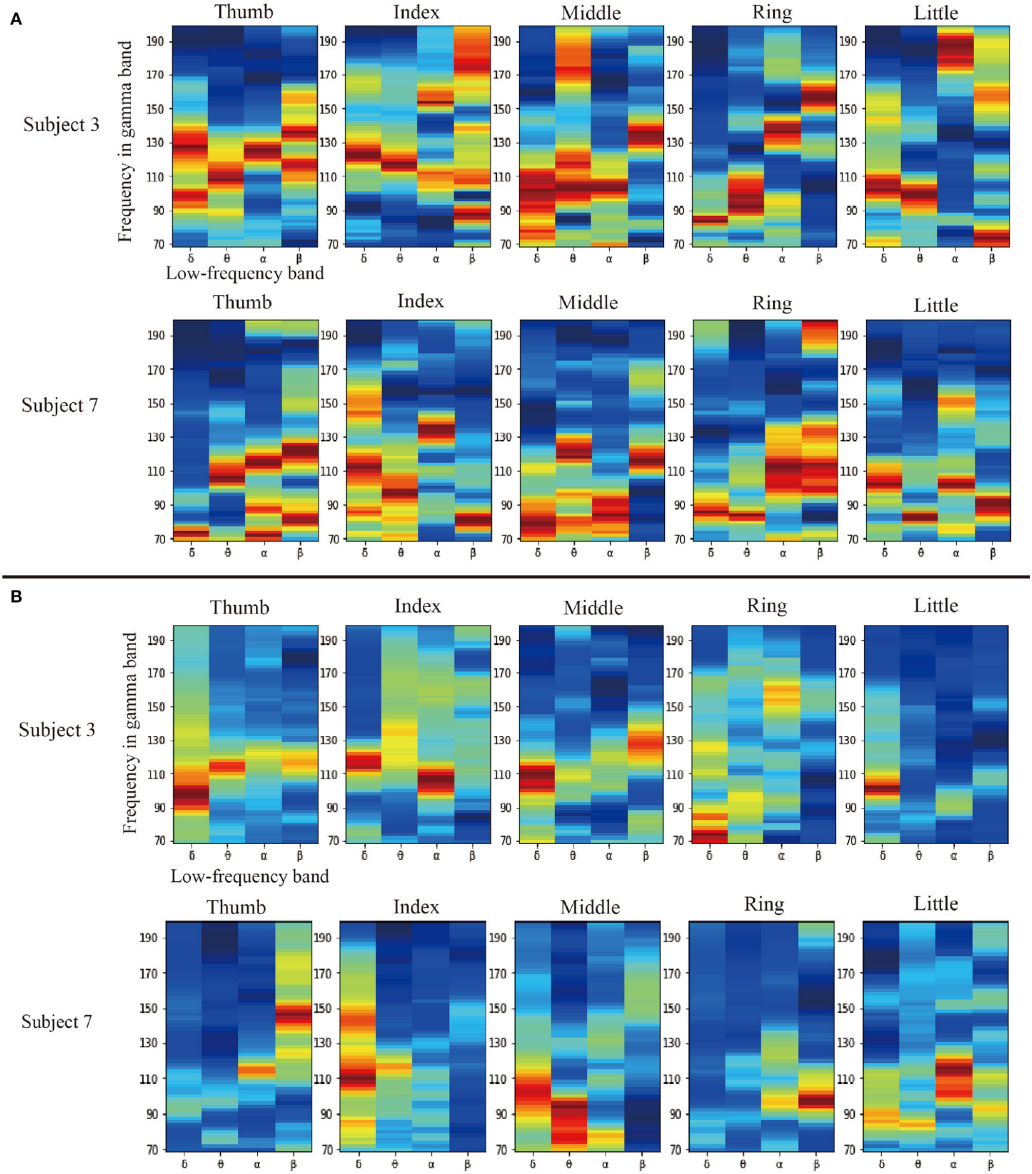
Comparison diagram of filter weight on two subjects in the fingerflex dataset, **(A, B)**, are in motion and rest states, respectively. In each sub-graph, the vertical axis represents the high-frequency in the high-gamma band, and the horizontal axis represents the four low-frequency bands: delta, theta, alpha, and beta. There are four columns in **(A, B)**, and each column represents a subject. At the same time, we show the specific situation of each finger of the subject. There are five rows in **(A, B)**, and each row represents a finger.

Firstly, an aforementioned ablation experiment is designed to verify the effectiveness of band selection. This experiment compares the results of whether our filter is used. The results with PAC are better than those without PAC. Therefore, we believe that the filter is adequate. For further analysis, we visualized our data-driven filter selection weights. As shown in Figures 2A, B are in motion and rest states, respectively. In each sub-graph, the vertical axis represents the high frequency, and the horizontal axis represents the four low-frequency bands: delta, theta, alpha, and beta. Due to space limitations, we randomly selected two subjects in the fingerflex dataset for display. There are four columns in Figures 2A, B, and each column represents a subject. At the same time, we show the specific situation of each finger of the subject. There are five rows in Figures 2A, B, and each row represents a finger. In Figure 2A, we can find that in the condition of movement, each has had significant PAC weights in the low-frequency band, it shows that the motion between high-frequency and low-frequency signals have a significant coupling, but from the point of view of different subjects, not between subjects in the movement of high-frequency coupling is different, this also shows each subject's signals are unique. It can be found in Figure 2B that in the resting state, a small amount of PAC weight is generated in some low-frequency bands, which indicates that there is some coupling between low-frequency signals and high-frequency signals at rest. However, the performance of these coupling quantities is much less than that in motion. This phenomenon shows that our filter can find some influential strong coupling frequency bands in motion and filter them out, thus improving the decoding accuracy.

### 3.3. Analysis of channel selection

Array electrodes collect ECoG signals. The electrode of each channel not only has its temporal property but also has spatial property in its position. Electrodes spatial properties represent the actual active regions of the cerebral cortex in the activity, so the practical analysis of electrode spatial properties can help the understanding of the working mechanism of the brain, in other words, through the known brain work mechanism of electrode space attribute verifies the consistency, can reflect the decoder will make it successful in avoiding noise, and whether the truly valid signal is analyzed and decoded to achieve its target function.

**Figure 3:**
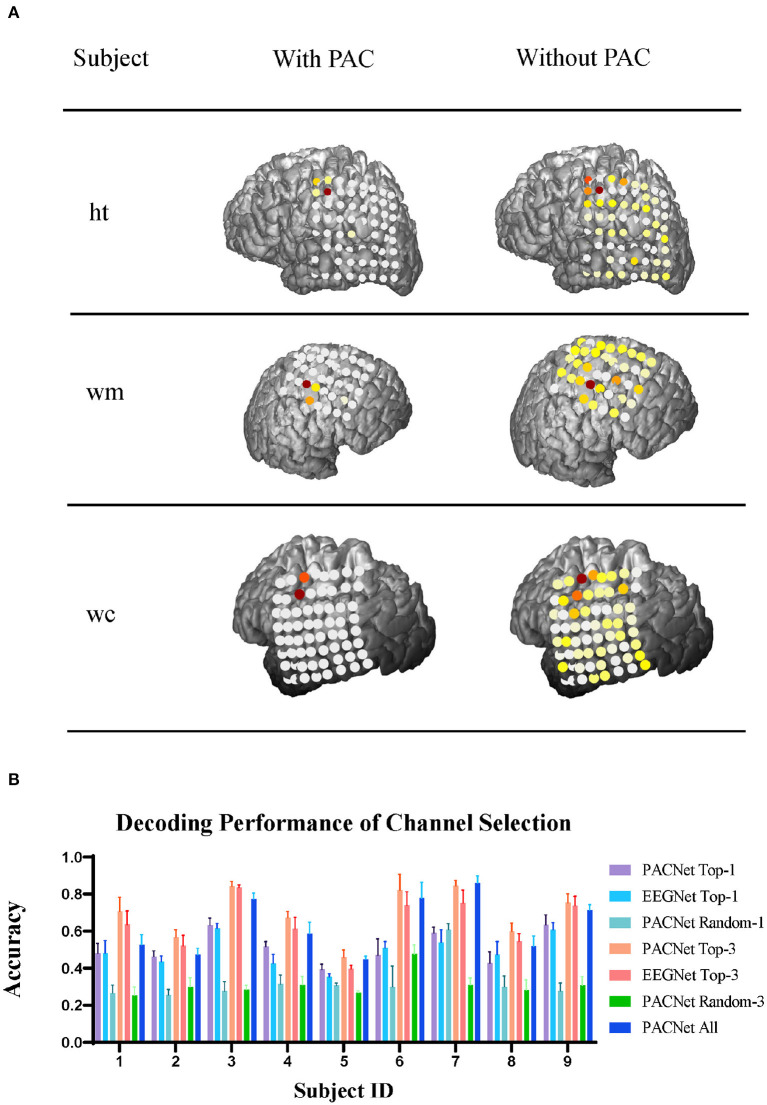
Channel contribution analysis: **(A)** To analyze three subjects in the ECoG electrodes channel contribution of visualization in the actual network model, the image is divided into two columns, the left is the use of our filter, filter is not used on the right is, network model after the training, each channel's contribution to the classification results of different weights are obtained. **(B)** Our electrode with high contribution for our elected the decoding performance tests of different method.

In this case, since we are using deep learning decoders, these decoders are somewhat uninterpretable initially, but as the technology develops, we can use the DeepLIFT algorithm (Schirrmeister et al., 2017) to interpret our model. We export the trained model in the motor dataset, calculate the contribution of each electrode's inputs through the DeepLIFT algorithm, and finally map it back to the actual brain model through rendering. As shown in Figure 3A, the right column is EEGNet, and the left column is our model. It can be seen intuitively that the model trained by our method can accurately hit the electrode in the motor cortex area near the central such of each subject. It is very concentrated, while the electrode activation distribution of the model trained by EEGNet is relatively scattered, and some electrodes on other cortexes are also activated, which may be one of the reasons for the low decoding performance of the decoder. The essence may lead the decoder to overfit some noises during the training step. To verify the actual validity of the channel contribution, we also designed a controlled experiment of 10-fold cross-validation on each subject in the fingerflex dataset, and the statistical analysis was conducted by paired *t*-test. As shown in Figure 3B, we tested the decoding performance of PACNet under different channel choices. We chose the top-1 and top-3 contribution channels and randomly selected 1 and 3 channels as controls for comparison. For further analysis, we add the test results of EEGNet using top-1 and top-3 channels from PACNet as a non-PAC comparison. Finally, we added the decoding performance of all channels as the baseline. It can be seen from Figure 3B that the selected top-1 and top-3 contribution channels are significantly better than randomly selected channels. In addition, top-3 contribution channels sometimes have higher decoding performance in baseline comparison, and the improvement of the decoding performance of Top-3 channels selection PACNet compared to EEGNet was significant (*p* = 0.0004).

## 4. Discussion

In this work, we propose an adaptive band filter that selects the proper frequency sub-bands in the high-gamma band in a data-driven way. Specifically, we leverage the phase-amplitude coupling (PAC) of the coupled working mechanism of synchronizing neuron and pyramidal neurons in neuronal oscillations, in which the phase of slower oscillations modulates the amplitude of faster ones, to help locate the fine frequency bands from the high-gamma range, in a task-specific and individual-specific way. Thus, the information can be more precisely extracted from ECoG signals to improve neural decoding performance. Based on this, an end-to-end decoder (PACNet) is proposed to construct a neural decoding application with adaptive filter banks in a uniform framework. The test results show two phenomena. On the one hand, when compared to the results in this, in which the input of EEGNet, DeepConvNet, and ShallowConvNet is the original raw ECoG recordings, We can see that these models cannot process a wide range of signals, this defect is evident in the test, so our method is very effective in dealing with this situation.

On the other hand, we can see the phenomenon in Tables 2, 3. When other methods show poor decoding performance, our method can sometimes show a considerable improvement in decoding performance. While other methods show better decoding performance than ours, the decoding performance of our method is almost the same as other methods. From this phenomenon, we propose a hypothesis that due to the uneven quality of ECoG signal acquisition, our method can eliminate the influence brought by these data with poor quality and much noise. The above gives us a new way of thinking, combining traditional methods with the latest machine learning methods, which may lead to some breakthroughs in current research. However, our approach is only a simple combination of PAC and EEGNet, which is a shortcoming of our approach. In the future, we will design a more integrated model to improve decoding performance better. In Section 3.3, compared to PAC and non-PAC methods, we can see that EEGNet can achieve a considerable performance improvement after data noise reduction. However, the performance of our method is still a little bit low. This phenomenon proved that our filter could help the deep learning decoders more accurately and automatically locate the electrodes on the corresponding cortex related to the task and achieve a better noising canceling effect to obtain higher decoding performance. It is an exciting discovery for us, and in future work, we will take advantage of this feature to make the decoder more automatic and precise.

## 5. Conclusion

This paper proposes a new adaptive band filter driven by phase-amplitude coupling for ECoG signals. We also propose a new general decode pipeline for current decoders to fit. Our method can use the existing mechanism of neuronal activity in the brain to dynamically calibrate the influential band in an unsupervised manner without a large number of data tags and can simply be combined with the existing classifier to improve the performance of the entire decoder. From the experiments in Section 3, we can conclude that our filter can help the deep learning decoders more accurately and automatically locate the electrodes on the corresponding cortex related to the task and achieve a better noise-canceling effect to obtain higher decoding performance, and to toward the big vision of cyborg intelligence (Wu et al., 2013).

## Data availability statement

The original contributions presented in the study are included in the article/supplementary material, further inquiries can be directed to the corresponding author.

## Author contributions

JL designed the methods and experiments, conducted the experiments, performed data analysis, made figures, and wrote the paper. YQ designed the experiments, made figures, and wrote the paper. GP supervised the projects. All authors contributed to the article and approved the submitted version.

## References

[B1] Aru J. Aru J. Priesemann V. Wibral M. Lana L. Pipa G.. (2015). Untangling cross-frequency coupling in neuroscience. Curr. Opin. Neurobiol. 31, 51–61. 10.1016/j.conb.2014.08.002 25212583

[B2] Bleichner M. Freudenburg Z. Jansma J. Aarnoutse E. Vansteensel M. Ramsey N. (2016). Give me a sign: decoding four complex hand gestures based on high-density ECoG. Brain Struct. Funct. 221, 203–216. 10.1007/s00429-014-0902-x 25273279 PMC4720726

[B3] Bragin A. Jandó G. Nádasdy Z. Hetke J. Wise K. Buzsáki G. (1995). Gamma (40-100 Hz) oscillation in the hippocampus of the behaving rat. J. Neurosci. 15, 47–60. 10.1523/JNEUROSCI.15-01-00047.1995 7823151 PMC6578273

[B4] Branco M. P. Freudenburg Z. V. Aarnoutse E. J. Bleichner M. G. Vansteensel M. J. Ramsey N. F. (2017). Decoding hand gestures from primary somatosensory cortex using high-density ECoG. NeuroImage 147, 130–142. 10.1016/j.neuroimage.2016.12.004 27926827 PMC5322832

[B5] Bruns A. Eckhorn R. (2004). Task-related coupling from high-to low-frequency signals among visual cortical areas in human subdural recordings. Int. J. Psychophysiol. 51, 97–116. 10.1016/j.ijpsycho.2003.07.001 14693360

[B6] Canolty R. T. Edwards E. Dalal S. S. Soltani M. Nagarajan S. S. Kirsch H. E.. (2006). High gamma power is phase-locked to theta oscillations in human neocortex. Science 313, 1626–1628. 10.1126/science.1128115 16973878 PMC2628289

[B7] Canolty R. T. Knight R. T. (2010). The functional role of cross-frequency coupling. Trends Cogn. Sci. 14, 506–515. 10.1016/j.tics.2010.09.001 20932795 PMC3359652

[B8] Canolty R. T. Soltani M. Dalal S. S. Edwards E. Dronkers N. F. Nagarajan S. S.. (2007). Spatiotemporal dynamics of word processing in the human brain. Front. Neurosci. 1, 14. 10.3389/neuro.01.1.1.014.2007 18982128 PMC2518055

[B9] Chakrabarti S. Sandberg H. M. Brumberg J. S. Krusienski D. J. (2015). Progress in speech decoding from the electrocorticogram. Biomed. Eng. Lett. 5, 10–21. 10.1007/s13534-015-0175-1

[B10] Chestek C. A. Gilja V. Blabe C. H. Foster B. L. Shenoy K. V. Parvizi J.. (2013). Hand posture classification using electrocorticography signals in the gamma band over human sensorimotor brain areas. J. Neural Eng. 10, 026002. 10.1088/1741-2560/10/2/026002 23369953 PMC3670711

[B11] Combrisson E. Nest T. Brovelli A. Ince R. A. Soto J. L. Guillot A.. (2020). Tensorpac: an open-source python toolbox for tensor-based phase-amplitude coupling measurement in electrophysiological brain signals. PLoS Comput. Biol. 16, e1008302. 10.1371/journal.pcbi.1008302 33119593 PMC7654762

[B12] Lawhern V. J. Solon A. J. Waytowich N. R. Gordon S. M. Hung C. P. Lance B. J. (2018). EEGNet: a compact convolutional neural network for EEG-based brain-computer interfaces. J. Neural Eng. 15, 056013. 10.1088/1741-2552/aace8c 29932424

[B13] Li Y. Zhang S. Jin Y. Cai B. Controzzi M. Zhu J.. (2017). Gesture decoding using ECoG signals from human sensorimotor cortex: a pilot study. Behav. Neurol. 2017, 3435686. 10.1155/2017/3435686 29104374 PMC5605870

[B14] Miller K. J. (2019). A library of human electrocorticographic data and analyses. Nat. Hum. Behav. 3, 1225–1235. 10.1038/s41562-019-0678-3 31451738

[B15] Miller K. J. Hermes D. Honey C. J. Hebb A. O. Ramsey N. F. Knight R. T.. (2012). Human motor cortical activity is selectively phase-entrained on underlying rhythms. PLoS Comput. Biol. 8, e1002655. 10.1371/journal.pcbi.1002655 22969416 PMC3435268

[B16] Miller K. J. Hermes D. Honey C. J. Sharma M. Rao R. P. Den Nijs M.. (2010a). Dynamic modulation of local population activity by rhythm phase in human occipital cortex during a visual search task. Front. Hum. Neurosci. 4, 197. 10.3389/fnhum.2010.00197 21119778 PMC2990655

[B17] Miller K. J. Schalk G. Fetz E. E. Den Nijs M. Ojemann J. G. Rao R. P. (2010b). Cortical activity during motor execution, motor imagery, and imagery-based online feedback. Proc. Natl. Acad. Sci. 107, 4430–4435. 10.1073/pnas.0913697107 20160084 PMC2840149

[B18] Miller K. J. Schalk G. Hermes D. Ojemann J. G. Rao R. P. (2016). Spontaneous decoding of the timing and content of human object perception from cortical surface recordings reveals complementary information in the event-related potential and broadband spectral change. PLoS Comput. Biol. 12, e1004660. 10.1371/journal.pcbi.1004660 26820899 PMC4731148

[B19] Miller K. J. Zanos S. Fetz E. Den Nijs M. Ojemann J. (2009). Decoupling the cortical power spectrum reveals real-time representation of individual finger movements in humans. J. Neurosci. 29, 3132–3137. 10.1523/JNEUROSCI.5506-08.2009 19279250 PMC6666461

[B20] Pan G. Li J.-J. Qi Y. Yu H. Zhu J.-M. Zheng X.-X.. (2018). Rapid decoding of hand gestures in electrocorticography using recurrent neural networks. Front. Neurosci. 12, 555. 10.3389/fnins.2018.00555 30210272 PMC6119703

[B21] Pilcher W. Rusyniak W. (1993). Complications of epilepsy surgery. Neurosurg. Clin. N. Am. 4, 311–325. 10.1016/S1042-3680(18)30597-7 8467217

[B22] Qi Y. Liu B. Wang Y. Pan G. (2019). “Dynamic ensemble modeling approach to nonstationary neural decoding in brain-computer interfaces,” in Advances in Neural Information Processing Systems, Vol. 32 (Vancouver, BC). Available online at: https://proceedings.neurips.cc/paper_files/paper/2019/file/3f7bcd0b3ea822683bba8fc530f151bd-Paper.pdf

[B23] Qi Y. Zhu X. Xu K. Ren F. Jiang H. Zhu J.. (2022). Dynamic ensemble bayesian filter for robust control of a human brain-machine interface. IEEE Trans. Biomed. Engg. 69, 3825–3835. 35700258 10.1109/TBME.2022.3182588

[B24] Schirrmeister R. T. Springenberg J. T. Fiederer L. D. J. Glasstetter M. Eggensperger K. Tangermann M.. (2017). Deep learning with convolutional neural networks for EEG decoding and visualization. Hum. Brain Mapp. 38, 5391–5420. 10.1002/hbm.23730 28782865 PMC5655781

[B25] Voytek B. D'Esposito M. Crone N. Knight R. T. (2013). A method for event-related phase/amplitude coupling. Neuroimage 64, 416–424. 10.1016/j.neuroimage.2012.09.023 22986076 PMC3508071

[B26] Voytek B. Secundo L. Bidet-Caulet A. Scabini D. Stiver S. I. Gean A. D.. (2010). Hemicraniectomy: a new model for human electrophysiology with high spatio-temporal resolution. J. Cogn. Neurosci. 22, 2491–2502. 10.1162/jocn.2009.21384 19925193 PMC2888789

[B27] Wang Y. Lin K. Qi Y. Lian Q. Feng S. Wu Z.. (2018). Estimating brain connectivity with varying-length time lags using a recurrent neural network. IEEE Trans. Biomed. Engg. 65, 1953–1963. 29993397 10.1109/TBME.2018.2842769

[B28] Wolpaw J. R. Birbaumer N. McFarland D. J. Pfurtscheller G. Vaughan T. M. (2002). Brain-computer interfaces for communication and control. Clin. Neurophysiol. 113, 767–791. 10.1016/S1388-2457(02)00057-3 12048038

[B29] Wu Z. Pan G. Zheng W. (2013). Cyborg intelligence. IEEE Intell. Syst. 28, 31–33.

[B30] Xie Z. Schwartz O. Prasad A. (2018). Decoding of finger trajectory from ECoG using deep learning. J. Neural Eng. 15, 036009. 10.1088/1741-2552/aa9dbe 29182152

[B31] Yanagisawa T. Hirata M. Saitoh Y. Goto T. Kishima H. Fukuma R.. (2011). Real-time control of a prosthetic hand using human electrocorticography signals. J. Neurosurg. 114, 1715–1722. 10.3171/2011.1.JNS101421 21314273

[B32] Zar J. H. (1999). Biostatistical Analysis. India: Pearson Education India.

[B33] Zhu Y. Li C. Jin H. Sun L. (2021). Classifying motion intention of step length and synchronous walking speed by functional near-infrared spectroscopy. Cyborg. Bionic. Syst. 2021, 9821787. 10.34133/2021/9821787 36285128 PMC9494720

